# Comment: pregnancy after bariatric surgery – achievements and open questions

**DOI:** 10.1186/s12884-023-05858-1

**Published:** 2023-08-02

**Authors:** Christian S. Göbl, Michael Feichtinger, Wolfgang Henrich

**Affiliations:** 1 Department of Obstetrics and Gynaecology, Medical University of Vienna, Vienna, Austria; 2 Wunschbaby Institut Feichtinger, Vienna, Austria; 3 Clinic of Obstetrics, Charité-Universitätsmedizin Berlin, Corporate Member of Freie Universität Berlin, Humboldt-Universität zu Berlin, Berlin Institute of Health, Berlin, Germany

**Keywords:** Bariatric surgery, Pregnancy, Gastric bypass, Sleeve gastrectomy, Obesity

## Abstract

Bariatric surgery confers potential advantages for obese patients, but also risks for pregnancy. Perinatal outcomes may be varying between surgical procedures. This topic was recently addressed by a systematic review in *BMC Pregnancy and Childbirth*. This commentary will discuss the scientific background and implications for future research.

## Background

Maternal obesity is a major health care issue, associated with several comorbidities in women at childbearing age, including infertility and a higher degree of insulin resistance. In pregnancy, obesity is associated with preeclampsia, pregnancy loss and the development of large for gestational age (LGA) infants. While obesity is closely related to gestational diabetes mellitus (GDM) or pregestational diabetes, there is emerging evidence that fetal overgrowth can also occur independently of maternal hyperglycaemia [1]. Consequently, the importance of adequate balanced nutrition increases further, and deviation from optimal nutrition leads to additional complications related to obesity.

Bariatric surgery is an established treatment for severe obesity, especially in the presence of metabolic co-morbidities [2]. For example, weight loss surgery was shown to be markedly more effective than intensive medical therapy alone to decrease and in some cases even to resolve hyperglycaemia in patients with type 2 diabetes, whereby gastric bypass (as a malabsorptive and restrictive procedure) and sleeve gastrectomy (as a restrictive procedure) showed durable weight loss and glycaemic improvements [3]. In younger women, weight loss after surgery improved periconception health, such as menstrual cycle irregularities and thus reduced obesity related subfertility [4]. As a consequence, the number of post bariatric surgery pregnancies and deliveries is increasing over the past decades [5].

In general, pregnancy after weight loss surgery is most probably safer than pregnancy in obese condition, however, it also poses a number of challenges and risks that need to be addressed [6]: Previous studies, including our own research, indicated intrauterine growth delay associated with history of bariatric surgery and especially with malabsorptive techniques [7, 8]. This type of “abnormal” (i.e. not genetically determined) development of small for gestational age (SGA) infants is most probably a consequence of nutritional deficiencies, as comparable findings were reported in offspring of mothers with anorexia nervosa, but this also occurs in developing countries in which a vast proportion of pregnant patients are undernourished [9]. Despite a reduction in GDM incidence, glucometabolic alterations are still present in mothers after gastric bypass, who showed high glycaemic variability with early postprandial hyperglycaemia followed by hypoglycaemic episodes at two to three hours after glucose ingestion [10, 11]. The increased glycaemic variability may be associated with fetal development, especially decreased fetal growth, as indicated by a recent study using continuous glucose monitoring in mothers after Roux-Y gastric bypass (RYGB) [12]. However, comparisons of different surgical procedures with focus on fertility and pregnancy outcomes are of major importance for recommending and choosing the best surgical approach in reproductive aged obese women.

## Discussion of the paper

This topic was recently addressed by Kistner *et al*., who published a systematic review of adverse perinatal outcomes after RYGB vs. sleeve gastrectomy, aiming to assess the incidence of pregnancy associated complications such as delivery of SGA or LGA infants [13]. Thereby, the authors specifically focused on studies with mean time to conception of less than four years and found a trend towards a higher incidence of both LGA and SGA infants in mothers after sleeve gastrectomy as compared to those with history of RYGB. This is in contrast to another systematic review with less stringent inclusion criteria, which indicated a markedly increased risk of SGA in mothers of RYGB and biliopancreatic diversion (another malabsorptive procedure), whereas this was not observed in mothers who underwent restrictive surgical procedures [8]. Likewise, the risk of LGA was notably reduced in mothers following RYGB (or biolopancreatic diversion), whereas only a tendency towards a lower risk for LGA was observed for women after laparoscopic gastric banding or sleeve gastrectomy in this study [8]. Comparable findings were observed by another recent network meta-analysis, indicating that RYGB resulted in a greater decrease in LGA but increased SGA infants as compared to sleeve gastrectomy [14]. The observed discrepancy between the systematic reviews may be explained by the stringent inclusion criteria used by the systematic review of Kistner *et al*., especially the rather short surgery to conception interval of four years. This was necessary as this study primarily focused on perinatal outcomes in the few years after weight loss surgery. Timing of pregnancy is indeed an important issue and it is generally recommended to avoid pregnancy for 12 to 18 months after surgery, when weight loss and nutritional status may become more stable [15]. Of note, women after sleeve gastrectomy showed a shorter surgery to conception interval in the recent publication by Kistner *et al*., what may be the reason for the (unexpected) high prevalence of SGA reported in the included studies.

## Conclusions

Bariatric surgery and its implications for pregnancy and childbirth is gaining increasing importance in the field of obstetrics and feto-maternal medicine. However, there are still a number of open questions. Further studies are required to clarify the best surgical technique and optimal time to conception interval for younger women at reproductive age. Fetal outcomes and especially long-term implications for the offspring including metabolic and neurological development need to be addressed. In any case, bariatric surgery in women at childbearing age requires careful planning of pregnancy and management by experienced obstetricians to offer optimal preconceptional and prenatal care. Future research is required targeting specific nutritional programs for patients to optimize maternal and neonatal outcomes.

## Data Availability

N/A.

## Abbreviations

GDM: Gestational Diabetes Mellitus
LGA: Large for Gestational Age Offspring
RYGB: Roux-Y Gastric Bypass
SGA: Small for Gestational Age Offspring
